# The implementation of the paediatric footwear program at community health level

**DOI:** 10.1186/1757-1146-4-S1-P29

**Published:** 2011-05-20

**Authors:** Alicia James, Cylie Williams, Christine Pappon

**Affiliations:** 1 Southern Health Community Services, Australia

## Background

Children spend around 30 hours a week in their school shoes - that's in excess of 15,000 hours during their school years. Correctly fitted shoes help reduce the risk of injury, improve comfort and assist in gross motor development and participation. Recent research indicates that foot deformities in children who wear incorrectly fitting footwear are increased, following the closure of a local shoe store and parents relating to podiatry staff of the financial difficulties of providing appropriate footwear, the podiatrist developed a quality project. The aim of the project was to provide cost effective and safe footwear for children that attend the services of Greater Dandenong and Cardinia Casey Community Health.

## Methods

200 local school children were assessed and 80 were determined to be wearing inappropriate fitting footwear. Bata footwear based in Mornington was approached and agreed to support the footwear program in the provision of footwear at a reduced cost for community health clients.

## Results/conclusions

The paediatric footwear program in the first 4 months has fitted over 120 children, with a follow-up qualitative survey to be conducted over the following months. Parents have welcomed this service and have regularly reported learning more about correctly fitting shoes and this service provision a welcome financial relief during hardship.

